# Supplementary material for: Using ELP Repeats as a Scaffold for De Novo Construction of Gadolinium-Binding Domains within Multifunctional Recombinant Proteins for Targeted Delivery of Gadolinium to Tumour Cells
Source: Int J Mol Sci. 2022 Mar 18;23(6):3297. doi: 10.3390/ijms23063297 (PMC8949254; doi:10.3390/ijms23063297)
Supplement: Supplementary file 1 [file ijms-23-03297-s001.zip › ijms-1616767-supplementary.pdf]

Table S1

Name	Sequence 5'→3'	Size	Restriction site
**E(Y)-for**	gtcccaggctatggcgtgccaggttctggt	30	
**E(Y)-rev**	tggcacgccatagcctgggac	21	
**M**	gacaaagacggtgacggcaccatcaccaccaaagaactg	39	
**EME**	acccggaaccagttctttggtggtgatggtgccgtcaccgtctttgtcaccagaacc	57	
**Enew**	gttccgggttctggtgtgccaggcagtggtgtacctgggtcaggcgttccgggttctggt	60	
**E2new**	cggaacgcctgacccaggtacaccactgcctggcacaccaga	42	
**M***	gacaaagacggtgacggcacgatcacgacgaaggagtta	39	
**EME***	taactccttcgtcgtgatcgtgccgtcaccgtctttgtcaccagaacc	48	
**Bgl-E(Y)**	gcatcagatctgtcccaggctatggcg	27	BglII
**Bam-M***	gcatcggatcctaactccttcgtcgtgatcgt	32	BamHI
**End-for**	gatctgttccgggttctggtgtgcctggtagcggtg	36	
**End-rev**	gatccaccgctaccaggcacaccagaacccggaaca	36	
**pJET1.2 forward sequencing primer**	cgactcactatagggagagcggc	23	
**pJET1.2 reverse sequencing primer**	aagaacatcgattttccatggcag	24	
**F3-1-dir**	aaagacgagccgcagcgtcgctccgcccgcctcagcgccaaaccggcaccgccaaaaccc	60	
**F3-1-rev**	tttttggctggcgctttttcggctttggctcgggttttggcggtgccggtttggcgctgag	63	
**FL-dir**	ccgaaaaaagcgccagccaaaaaaggtggtggtggttctggtggcggcggctctgg	56	
**Bam-L-rev**	gcatcggatccaccaccagagccgcc	26	BamHI
**R1-for**	gcggttaccggtcgtggtgatagcggttctgg	32	
**R1-rev**	accagagccgctgcccgaaccagaaccgctatcacc	36	
**Bgl-R1-for**	gcatcagatctgcggttaccggtcg	25	BglII

**Bam-R1-rev**	gcactggatccaccagagccgctgc	25	BamHI
**W4 seq rev**	ggacacgctgaacttgtgg	19	
**Bgl-F-for**	gcatcagatctaaagacgagccgcag	26	BglII
**Eco-W4-rev**	gcactagaattcaccgctaccaggc	25	EcoRI
**Exp1 for**	acaacaccacaacaccatcatcatcatcatcatgg	35	
**Exp2 for**	accaccccacaacaccatcatcatcatcatcatgg	35	
**Exp rev**	catatgtatatctccttcttaaagttaaacaaaattatttctag	44	
**Exp1 seq for**	catatgacaacaccacaacacc	22	
**Exp2 seq for**	gaccaccccacaacacc	17	
**Eco-F-for**	gcatcgaattcaaagacgagccgcag	26	EcoRI
**Nco-F-for**	agcactccatgggtaaagacgagccgcagc	30	NcoI
**Nco-E-rev**	agcacaccatggaaccgctaccaggcacacc	31	NcoI
